# ILLUSTRATIONS OF EXTENDED WORKING LIVES: WHAT INSIGHTS DO THE CONCEPTS OF EXCLUSION AND PRECARITY OFFER?

**DOI:** 10.1093/geroni/igad104.1791

**Published:** 2023-12-21

**Authors:** Amanda Grenier

**Affiliations:** University of Toronto, Toronto, Ontario, Canada

## Abstract

Amanda Grenier will act as the discussant for the set of papers in this symposia session, bridging insights from European research with North American perspectives. Situated as an attempt to bridge research findings with a larger critical practice of theory-building theory, she will explore what the concepts of exclusion and precarity offer our interpretations of these papers, as well as how the findings of each contribute to the state of knowledge on exclusion and precarity. She will begin with a brief clarification of the definitions and conceptual boundaries of social exclusion and precarity carried out in Europe and North America and situate key findings of the papers as an effort to theorize older workers lives in contemporary conditions. For example, the well-known concept and domains of social exclusion can be considered to offer ways of understanding processes of exclusion at the mezzo level, as exercised through policies, practices, and/or place. Illustrations from the papers will be linked with existing work on exclusion. She will then draw attention to the concept of precarity as a lens of analysis to connect the micro-level vulnerabilities experienced in the working lives of older people with the shifting social and state structures that form the backdrop for older people’s lives (and the systems within which European research is carried out). Specific examples will be drawn from the set of papers, and a moderated question and answer period will follow.

